# Evaluation of *Platonia insignis* Mart. (Bacuri Butter) and Biopolymers from the Puree of *Allium cepa* L. (Yellow Onion Bulb) for Wound Healing in Horses

**DOI:** 10.3390/pharmaceutics16111457

**Published:** 2024-11-15

**Authors:** André M. Resende, Beatriz A. Miranda, Luiza B. Silva, Andressa B. Oliveira, Márcio B. Castro, Isabel L. Macêdo, Bruno S. L. Dallago, Hernane S. Barud, Marco A. Costa Borges, Clovis A. Ribeiro, Diogenes S. Dias, Rita C. Campebell

**Affiliations:** 1 Hospital Escola de Grandes Animais, Faculdade de Agronomia e Medicina Veterinária (FAV), Universidade de Brasília (UnB), Área Especial SRB, Galpão 4, Granja do Torto, Brasília 70636-200, DF, Brazil; andremresende27@gmail.com (A.M.R.); beatrizalvesdemiranda3@gmail.com (B.A.M.); luizabezerra.vet@gmail.com (L.B.S.); andressa.vet20@gmail.com (A.B.O.); mbcastro@unb.br (M.B.C.); isabeluanamacedo@gmail.com (I.L.M.); dallago@unb.br (B.S.L.D.); 2 Laboratório de Biopolímeros e Biomateriais (BIOPOLMAT), Departamento de Química, Universidade de Araraquara (UNIARA), Araraquara 14801-320, SP, Brazil; hsbarud@uniara.edu.br (H.S.B.); odontomacb@yahoo.com.br (M.A.C.B.); 3 Departamento de Química Analítica, Fisico-Química e Inorgânica, Instituto de Química, Universidade Estadual Paulista Júlio de Mesquita Filho (UNESP), Araraquara 14800-060, SP, Brazil; clovis.ribeiro@unesp.br (C.A.R.); digignes@gmail.com (D.S.D.)

**Keywords:** bacuri butter, onion biopolymer, horses, wounds healing

## Abstract

**Background/Objectives:** Skin injuries are common in the equine clinical practice, requiring effective treatment to support natural healing. Bacuri butter is gaining attention for its potential in wound healing and its anti-inflammatory, antimicrobial, and antioxidant properties. Natural polymers such as onion (*Allium cepa*) bioplastics have been investigated for their potential as occlusive dressings and for tissue regeneration. **Methods:** This study evaluated the healing process of experimentally induced skin wounds on horses treated with bacuri butter, washed onion film, and unwashed onion film. Clinical and histopathological analyses of the wounds were conducted in six clinically healthy horses over 28 days, with a control group receiving Ringer’s lactate solution. The onion films were produced and characterized for their chemical structure and properties, while the bacuri butter was sourced and prepared for application. **Results:** All treatments, including the control group, promoted wound healing without relevant differences in wound contraction rates, gross aspect, or histopathological parameters. **Conclusions:** Therefore, despite minor variations observed in the clinical evaluations between the treatment groups, the bacuri butter or onion biopolymer showed no significant healing effect on skin wounds in horses. Additionally, this study showed the potential of equine models in testing novel therapeutic approaches for wound healing, benefiting both veterinary and human medicine.

## 1. Introduction

Skin injuries are among the most frequent occurrences in equine practice due to the species’ active behavior and intrinsic reactions. Although many alternative treatments are known for managing skin wounds, the selected method must provide a favorable environment, allowing for natural healing progression and not delaying the repair process [1]. Herbal medicines have been routinely tested as an alternative treatment in large animal surgical practice due to their effective effects on the wound healing process [2,3,4].

There has been a growing interest in bacuri seeds in herbal medicine. In rural areas, the oil extracted from the seeds is called “bacuri butter”, which, when heated, becomes solubilized and is widely used in treating various dermatoses and wound healing in animals [5,6,7]. Alternative wound treatments have been evaluating natural polymers for promoting healing and tissue regeneration, as they can maintain a controlled microenvironment at the injury site [8,9]. In this context, onion (*Allium cepa*), is one of the most widely studied plant species from the food industry in recent years due to its beneficial components.

The onion constitutes proteins, fibers, and mineral salts, and has been utilized in the production of bioplastics through hydrothermal treatment of its pulp [10].

The onion’s medicinal and biopolymer properties as occlusive dressings hold promise for wound treatment. Given the increasing demand for bioplastics from renewable sources across various applications, research efforts have been directed towards environmental preservation. Consequently, a biodegradable onion film (*Allium cepa* L.) was developed, encompassing the characterization of its chemical structure, production protocol, and properties [11]. 

Due to similarities between the normal wound healing processes in humans and horses and the challenges imposed by chronic wounds in both species, horses are physiologically relevant models for investigating the mechanisms underlying wound repair. Moreover, horses have been proposed as ideal models for testing novel therapeutic approaches, potentially benefiting both veterinary and human medicine [12]. 

This study assessed the healing process of experimentally induced skin wounds on the backs of horses treated with bacuri butter, washed onion film, and unwashed onion film. The clinical and histopathological aspects of healing were evaluated and compared to the control group which were cleaned with lactated Ringer’s solution.

## 2. Materials and Methods

### 2.1. Animals

The animal study protocol was approved by the Ethics Committee on the Use of Animals (CEUA) of the Universidade de Brasília-UnB, Brasília, Brazil (Protocol No. 23106.064222/2021-19, on 12 July 2021), regulated by the National Council of Ethics. Animals used in this experiment were abandoned in public places and gathered by the Department of Agriculture of the Federal District (Brasília). All horses received clinical care, vaccines, deworming, and adequate nutrition until they were healthy and fit to participate in the research. All procedures were under animal welfare protocols, and all horses rested for approximately 35 to 40 days to fully heal wounds. Thus, all animals used in the experiment were donated to new owners.

Six clinically healthy horses were selected. Five females and one castrated male, of which, five were of no defined breed, and one was a Brazilian equestrian (BH), with a body weight average of 321.6 kg, were submitted to clinical examinations and hematology and serum biochemistry evaluation (urea, creatinine, gamma-glutamyl transferase, aspartate aminotransferase, and albumin) with unremarkable results. Deworming was performed orally with ivermectin and praziquantel (Padock Plus NF, CEVA, Paulína, SP, Brazil) in addition to medication with a prophylactic dose of antitetanic serum (Venco-sat, Vencofarma, Londrina, PR, Brazil). All horses were kept in stalls and maintained throughout the experiment in good hygiene conditions, fed with commercial feed (Nutrina Equinos Premium, Nutrina Rações e Minerais, Brasília, DF, Brazil) and coast cross hay, and water ad libitum. The heart rate (HR), respiratory rate (RR), mucous membranes, capillary refill time (CRT), rectal temperature (T°C), intestinal motility, attitude, and appetite were evaluated daily during the experiment.

### 2.2. Biopolymers from Allium cepa L. (Onion Bulb)

**Figure 1:**
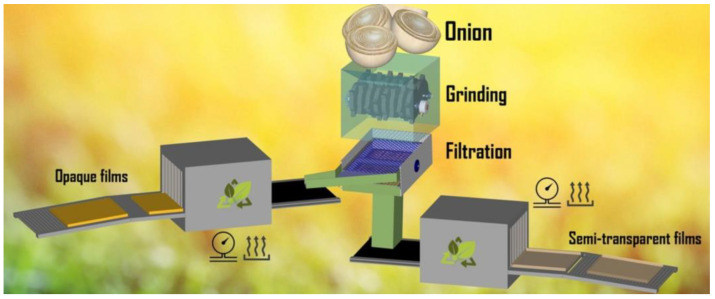
The onion film production scheme [11].

**Figure 2:**
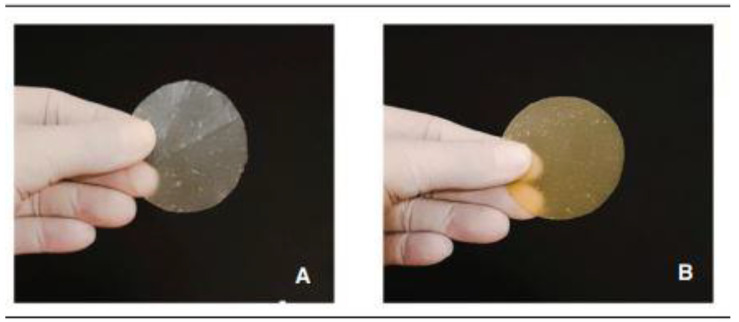
(**A**) The onion films from the washed hydrothermally treated pulp (W-HTP) and (**B**) unwashed hydrothermally treated pulp [10].

The onion biopolymers were supplied by the Biopolymers and Bio-materials laboratory (BIOPOLMAT), Department of Chemistry, University of Araraquara (UNIARA), Araraquara, SP, Brazil. They were processed and treated hydrothermally and not washed (HTP) or treated hydrothermally and washed (W-HTP). The HTP film contains both soluble and insoluble carbohydrates, whereas the W-HTP film lacks soluble carbohydrates and contains fewer insoluble carbohydrates compared to the HTP film [10]. This study assessed whether these compositional differences affected the healing process. The onion films were produced from yellow-type bulbs (*Allium cepa* L.), which were medium in size and with a mean moisture content of around 89%. Prior to use in treatment, the onions were washed with water to remove impurities. Afterward, the bulbs were cut lengthwise into four pieces and rewashed [10]. Then, the onion bulbs were placed in an industrial blender (800 W, 4 L (FAK Itajobi Indústria Metalúrgica, Itajobi, SP, Brazil), filtered through qualitative filter paper (Grade 292, Boeco qualitative filter, Hamburg, Germany), and then washed with distilled water ten times to eliminate the characteristic odor. HTP was prepared with onion pulp that had been autoclaved for 30 min at 121 °C and 1.2 kgf/cm^2^, ground in an Ultra Turrax disperser (IKA T18, Campinas, SP, Brazil) for 10 min at 7000 rpm, poured into 90 mm diameter Petri dishes, and dried in a fume hood at room temperature with air circulation for 6-h or until the film detached. Similarly, W-HTP films were washed with distilled water and dried before grinding. The produced HTP and W-HTP (Figure 1 and Figure 2) were cut into squares of 3 cm on each side and packaged in surgical grade paper before being sent for sterilization in Co-60 gamma rays (using gamma energy of approximately 1.25 MeV with a dose rate of approximately 1.44 kGy/h), (Gammacell GC220 MDS Nordion irradiator, Laval, QC, Canada) with a dosage of 25 kGy at the Gamma Radiation Laboratory (GAMALAB) of the Department of Nuclear Energy at the Federal University of Pernambuco–Recife, PE, Brazil.

### 2.3. Bacuri Butter

**Figure 3:**
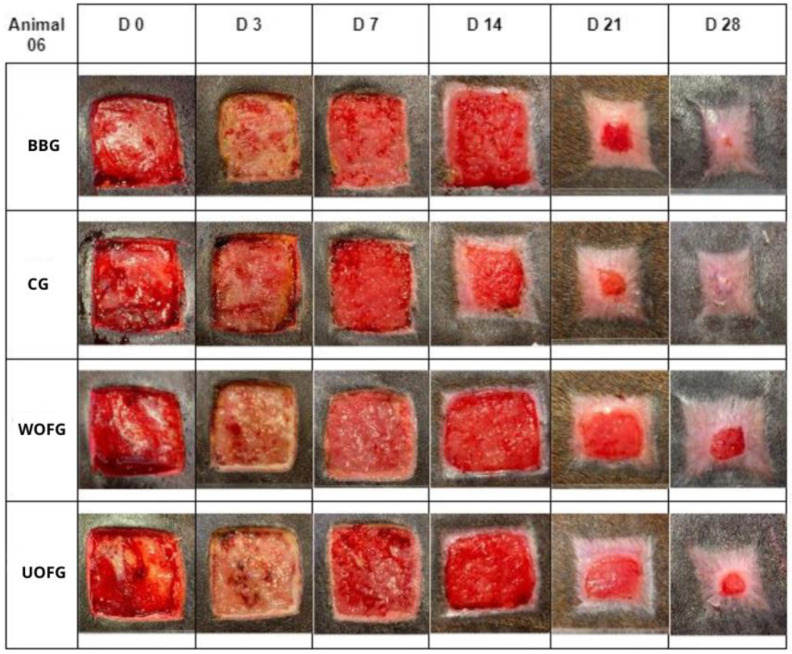
Photographic images of the wounds in the lumbar region of the horses: bacuri butter group (BBG), control group (CG), washed onion film group (WOFG), and unwashed onion film group (UOFG), on days 0 (D0), 3 (D3), 7 (D7), 14 (D14), 21 (D21), and 28 (D28).

Bacuri butter was supplied by the company Amazon Oil Industry (Figure 3). Bacuri (*Platonia insignis* Mart.) fruits were collected when ripe and were chosen for their intense yellow or orange color. The seeds were extracted and cleaned to remove any remaining pulp or skins, followed by a drying and maceration process to release the fats. The fat was extracted by grinding the seeds under pressure and heat, and filtered to remove impurities. The bacuri butter fat was manually mixed to reduce its thickness at room temperature, improving its solubility and enhancing its adhesion to the dressings [5].

### 2.4. Production of the Wounds

The horses underwent a 12-h food fast and a 6-h water fast prior to surgery. Tranquilization was performed using 10% xylazine hydrochloride (Equisedan, J.A. Saúde Animal, Patrocínio Paulista, SP, Brazil) in 1 mg × kg^−1^ intravenous dosage. Trichotomy was performed on both sides of the lumbar region, cranial to the thigh tuberosity, and ventral to the transverse processes of the L1-L6 lumbar vertebrae. Antisepsis was performed with degerming povidone-iodine and 70% alcohol. Local infiltrative anesthesia of the skin was performed with 2% lidocaine without a vasoconstrictor (Lidocaine hydrochloride 2%, Hipolabor Farmacêutica, Belo Horizonte, MG, Brazil), and a mold for the wounds was designed with a surgical pen. On both sides of the lumbar region, cranial to the sacrum and ventral to the transverse process of the lumbar vertebrae (L1–L6), skin incisions were made to remove four 3 × 3 cm square-shaped skin flaps, resulting in open wounds on each side, spaced 5 cm apart each from the skin to the subcutaneous tissue, and exposing the muscle fascia.

### 2.5. Treatments 

The horses were randomly selected for gross and microscopic evaluations. In three animals, the right back side was used for clinical wound assessment, and the left was used to perform biopsies for microscopic evaluation. In the other three horses, the reverse procedure was carried out. Treatment protocols were performed on the wounds in the craniocaudal direction, on both sides as follows: bacuri butter group (BBG), control group (CG), washed onion film group (WOFG-W-HTP), and unwashed onion film group (UOFG-HTP).

Clinical evaluation was conducted on the other horses’ back sides by photographing the identified wounds with a ruler. The daily treatment of the CG wounds included washing with Ringer’s lactate solution and bandaging with gauze. A layer of bacuri butter was applied to the BBG wounds, and the WOFG and UOFG groups received one film, covering each wound. The dressing of wounds followed the same pattern daily for the 28 days of the experiment for the CG and BBG groups. In the WOFG and UOFG groups, the wound dressings were applied only when the film was degraded entirely, or the wound area was left, with approximately 20 changes being made for each treatment. All wounds were cleaned with lactated Ringer’s solution before dressing, which included a gauze fixed with micropore tape/adhesive tape and finished with a cover (JHR Confecções, Brasília, DF, Brazil), covering the entire back and abdomen of the animals. All six horses received phenylbutazone in the dosage of 4.4 mg × kg^−1^ intravenously (SID) (Equipalazone, CEVA, Paulínia, SP, Brazil) immediately post-surgery and for the next three days to control pain and edema.

### 2.6. Clinical and Photographic Evaluation of the Wounds

Clinical and photographic evaluations were carried out since the day of the surgery (D0) and on the third (D3), seventh (D7), fourteenth (D14), twenty-first (D21), and twenty-eighth (D28) days after. In the clinical evaluation, the following parameters were observed: edema, hyperemia, local hemorrhage, crust, clot formation, the presence and type of exudate, granulation tissue, and epithelialization. The calculation of the wound area and the contraction rate were evaluated through photographic monitoring. The wound area was measured using Image J2 Software (Wayne Rasband National Institutes of Health, Bethesda, MD, USA, http://rsb.info.nih.gov/ij/download.html, accessed on 30 March 2023). The degree of wound contraction was calculated using the equation proposed by Ramsey and co-workers (1995) [13]: contraction rate (%) = 100 × (W0 − WA)/W0, where W0 represents the original wound area immediately after its production and WA represents the wound area at the evaluation time (0, 3, 7, 14, 21, or 28 days), expressed as a percentage.

### 2.7. Biopsy and Histopathological Evaluation 

After local infiltrative anesthesia with 1 mL of 2% lidocaine without a vasoconstrictor (Lidocaine hydrochloride 2%, Hipolabor Farmacêutica, Belo Horizonte, MG, Brazil), skin biopsies were performed with a 6 mm diameter punch, covering the boundaries of the wound and the intact tissue, on the day of surgery (D0) and the days D3, D7, D14, D21, and D28. The fragments were collected around the wound in a clockwise direction, ensuring that they were not taken from the sites of previous biopsies. Tissue samples were fixed in 10% buffered formalin, pH 7.0, and sent to the Veterinary Pathology laboratory at the University of Brasília. The biopsy samples were processed by progressive dehydration using a graded series of ethanol concentrations (70%, 95%, and 100%), followed by clearing with xylene and embedding in paraffin at 60 °C. The 4 μm thick sections were cut from the paraffin blocks using a rotary microtome, mounted on glass slides, deparaffinized in xylene, and stained with hematoxylin and eosin. Semiquantitative microscopic analyses were performed as follows: the inflammatory infiltrate of neutrophils, eosinophils, and monocytes; the healing process including fibroblast proliferation and neovascularization; and hemorrhage and skin ulceration. 

Skin samples from all animals on days 0, 7, and 28 were additionally evaluated using Masson’s trichrome staining (Histokit, EasyPath, Masson’s Trichrome–with aniline blue, Grupo Erviegas, Indaiatuba, SP, Brazil). The samples were assessed for fibroplasia (the amount of collagen fibers), the degree of reorganization of the collagen fibers (the amount of realigned collagen fibers), and vascularization. All pathological findings were classified as (−) absent, (+) mild, (++) moderate, or (+++) marked. 

### 2.8. Statistical Analysis

The collected data were subjected to normality analysis using the Shapiro–Wilk test. Then, the Kruskal–Wallis test was applied, considering the day of the experiment and the treatment (CG, BBG, WOFG, or UOFG) as fixed variables. The pairwise Wilcoxon tests were conducted to provide evidence of significant differences. Statistical analysis for histopathological results used Friedman’s chi-square test, which used animal (subject) as a block in a randomized complete block design. In addition, repeated measures analysis was performed using compound symmetry (CS) as a covariance structure. All analyses were performed using SAS software (v9.4, Cary, NC, USA), adopting a significance level of 5%.

## 3. Results

### 3.1. Physical Assessment

Clinical alterations in the physical assessments associated with surgical wounds on any day of the experiment were unremarkable in all horses. From D1 to D7, the wounds showed edema and sensitivity to pain, and the animals became more reactive during manipulation to apply dressings. However, physiological parameters remained within the normal ranges established for the species.

### 3.2. Assessment of the Wounds

On D1, the BBG wound grossly showed minor bleeding, moisture, and hyperemia compared to the other treatments. Hemorrhage was observed up to D3 in the CG wound, while the WOFG and UOFG wounds had evident blood clots and moist wound beds due to the partial degradation of the films. Regarding hemostasis, blood and clots were observed in the visual assessment until D7 in all groups.

During the D1 dressing, complete film degradation was observed in UOFG wounds, while only partial degradation of the films in WOFG wounds were observed. In both wounds, moisture and translucent exudate were observed, which, from D7 onwards, became yellowish. Between D3 and D14, granulation tissue was detected within the wounds, which were initially mild, then followed by an irregular and granular aspect, and bleeding on D7; on D14, it showed a smooth and uniform appearance throughout the entire wound length in most animals. In three animals, there was an exuberant granulation tissue in the BBG and CG wounds on D14, which progressed to reorganization, and similarly in the WOFG and UOFG wounds in two animals from D14 to D21.

In all wounds, the epithelialization of margins began between D14 and D21. On D21 and D28, the epithelialization margins were marked in the BBG and CG wounds, grossly compared to WOFG and UOFG wounds. The epithelialization process in the BBG wounds was evident between D14 and D21, especially in animals 5 and 6 (Figure 3).

#### 3.2.1. Wound Area

**Figure 4:**
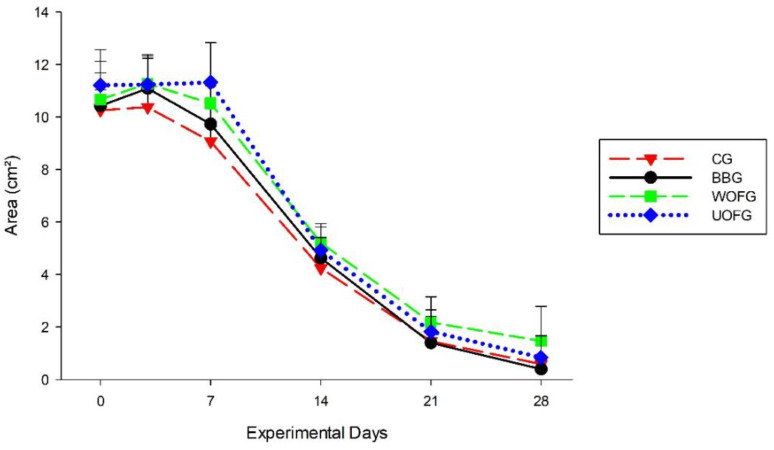
The mean wound areas, in cm^2^, of the lumbar region of the horses in the control group (CG) and the groups treated with bacuri butter (BBG), washed onion film (WOFG), and unwashed onion film (UOFG), on days 0 (D0), 3 (D3), 7 (D7), 14 (D14), 21 (D21), and 28 (D28).

On D7, statistical differences were observed between the groups’ mean wound areas, with the smallest area in the CG and the largest area in the UOFG. A progressive reduction in wound area was observed in all groups until D28 (Table 1 and Figure 4).

#### 3.2.2. Wound Contraction Rate

**Figure 5:**
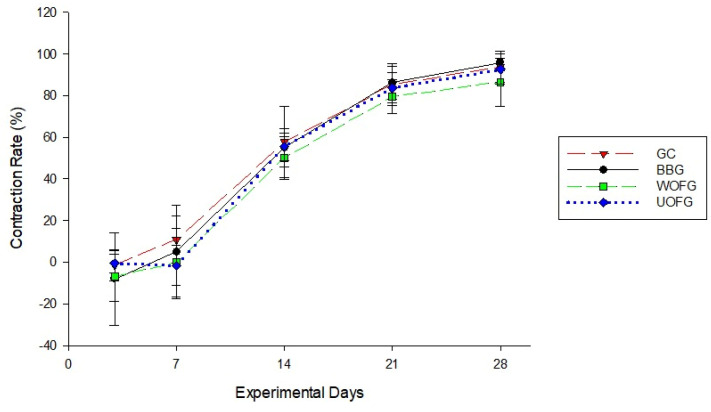
The mean contraction rates, in percentage, of the wounds of the horses in the control group (CG) and the groups treated with bacuri butter (BBG), washed onion film (WOFG), and unwashed onion film (UOFG), on days 3 (D3), 7 (D7), 14 (D14), 21 (D21), and 28 (D28).

No statistical differences in the wound contraction rates were observed between all of the groups on days seven and fourteen (Table 2 and Figure 5).

### 3.3. Histopathological Evaluation

No statistical differences existed between the groups in the histopathological evaluation or for any of the evaluated parameters (Table 3). On D21 and D28, there was a discrete infiltration of eosinophils in the CG, WOFG, and UOFG wounds. In addition, the mononuclear inflammatory infiltrate was moderate on D7 in the WOFG and UOFG wounds and on D21 in the CG wounds.

**Figure 6:**
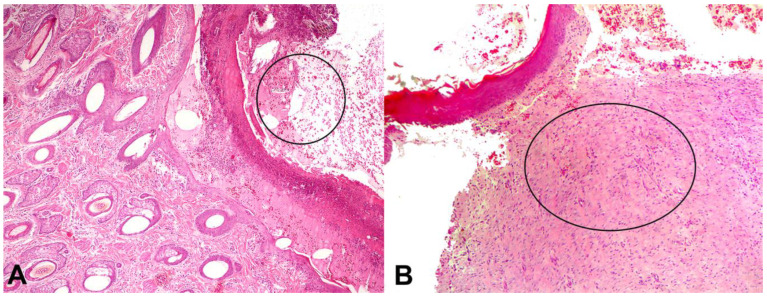
(**A**) Photomicrograph of the wound from animal 6 of the bacuri butter group (BBG) on D3, demonstrating edema, inflammatory cells, and cellular debris hyperkeratosis and cellular debris (circled area) (Obj. 4×, H&E). (**B**) WOFG, D28. Intense collagen deposition (fibroplasia) in the dermis (circled area) (Obj. 10×, H&E).

**Figure 7:**
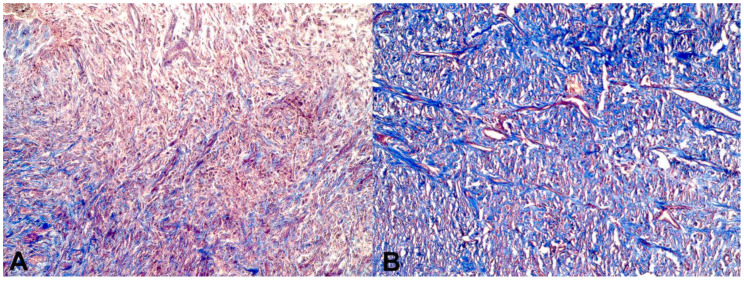
(**A**) Photomicrograph of the wound from animal 6 of the bacuri butter group (BBG). D7, mild fibroplasia and the reorganization of collagen fibers. (**B**) D28, intense fibroplasia and the reorganization of collagen fibers (Obj. 10×, Masson’s trichrome).

Fibroplasia (the formation/deposition of collagen fibers in the wound) was moderate from D7 onwards in the UOFG wounds, and on D14, it was intense in the other groups evaluated, except for the BBG wounds. From D21 onwards, all groups showed a marked fibroplasia process in all wounds (Figure 6A,B). On D7, neovascularization was mild in the WOFG wounds and moderate in the other groups. From D14 onwards, neovascularization was accentuated in all groups. Despite no differences detected, these findings characterized the evolution of the granulation tissue formation process in all of the skin wounds (Table 4, Figure 7).

## 4. Discussion

The standardization of experimentally induced wounds on the backs of horses is an essential procedure for better interpreting the local treatments used, as well as for evaluating the retraction and area of wounds. The size of the wounds established in the horses was similar to those performed in other studies [2,4,14,15], since the shape of these wounds does not affect healing time in horses [16].

Considering that humans and horses present similarities in the natural healing of wounds, horses are considered a physiologically relevant model, both for the study of the mechanisms involved in wound repair and for the evaluation of new therapies with possible applicability in the clinical routine of humans [12]. The skin of humans and horses also present similarities in terms of architecture, both comprising two main layers that protect the internal organs from mechanical damage and pathogen invasion. The epidermis has stratified squamous epithelial cells and a thick dermis in both horses and humans but shows sparse hair follicles in humans, while in horses, the hair follicles are densely distributed [17,18].

The administration of phenylbutazone within three days of the post-wound surgery provided comfort and pain reduction for the horses. Only a mild level of discomfort was observed during dressing changes on these postoperative days. The systemic administration of phenylbutazone was used in analogous investigations [15,19,20], possibly impacting all wounds in the control and experimental animals in this study. A similar discomfort during dressing changes in the horses on the initial days following the surgery was also noted in other studies [15,21].

The use of similar surgical wounds in the horses’ back for gross evaluation and biopsy collection mitigated any influence of the biopsies on the assessment of wound area and degree of wound contraction, as previously reported [2,14]. During the initial post-surgical week and within the inflammatory phase, edema, clots, hyperemia, and hemorrhage were observed in all animal groups, ranging from mild to severe and with more pronounced changes on day three and milder manifestations on day seven, consistent with previous reports [20].

The results for epithelialization revealed wounds with irregular edges and a raised, grainy center at the end of the experiment. This could be attributed to the prolonged daily application of bacuri butter, which may act as an exogenous irritant, potentially exacerbating chronic inflammation [5] and thereby contributing to the raised wound centers. In this study, significant differences in wound epithelialization were not observed between the CG and BBG wounds, contrary to those detected in another study using bacuri butter on wounds [3], and possibly attributed to the prevention of tissue dehydration, maintaining wound moisture, and stimulating epithelialization and angiogenesis.

Until D7, thin and easily removable clot crusts were observed in the recovering wounds. From the seventh day onwards, there was thickening and increased adherence of the crusts, detaching spontaneously from the fourteenth day onwards. Crusts were easily removed from the center of the wound on D21, indicating progression toward epithelization at the end of the experiment. Despite no significant differences, hemorrhaging and blood clots were detected up to D7 in the BBG wounds, suggesting a suitable containment of bleeding, followed by the UOFG, WOFG, and CG wounds, respectively.

The moisture observed in the wounds of the WOFG and UOFG groups from D1 to D21, likely more pronounced compared to other groups even during the partial degradation of the film, was possibly linked to the degradation properties of the unwashed onion biofilm. This maintained a more humid wound environment, likely due to the presence of soluble carbohydrates in its composition [11]. Moreover, the serofibrinous exudate observed until day 21 in most of the wound beds likely supported epithelial cell regeneration and migration. The exudate acted as a protective barrier against infection, supplied essential nutrients to activate cellular metabolism, and promoted healing [20,22]. In this experiment, the exudate decreased as granulation tissue progressively filled the wound.

The average wound areas showed differences between the groups on D7, possibly due to excessive exudation observed from D3 onwards. Excessive exudation is associated with an increased risk of colonization by wound surface microorganism, systemic infection, or persistent wound inflammation [23]. Moreover, excessive exudation can lead to maceration at the wound edges, impairing keratinocyte migration toward the center of the lesion and delaying epithelialization [24].

At the end of the experiment (D28), contrasting differences between the wound areas could not be proven in all groups. The pro-healing effect of bacuri seed butter (BBG) in horses could not be detected, as observed in a murine experimental cutaneous leishmaniasis study treating ulcerative lesions with topical formulations containing bacuri seed butter [25]. Similarly evident in the average wound areas, no significant differences were detected between the treatment groups for the wound contraction rate. The wound contraction rate may vary according to location, contracting at 0.8 to 1.0 mm/day on average in horses with defects of 400 cm^2^ in the flank region [26].

Histologically, no significant differences in the acute inflammatory process, fibroplasia, and the reorganization of collagen fibers (granulation tissue formation) within wounds were also demonstrated between the treatment groups. The healing, anti-inflammatory, antimicrobial, and antioxidant properties of bacuri butter, due to the fatty acids in its composition [5]; increase in granulation tissue formation; and the organization of collagen fibers [25] could not be shown in this study. Collagen synthesis begins as early as day two, peaking between days five and seven, and continues to increase for three weeks, enhancing wound strength. Collagen fibers tend to align parallel to the wound surface and perpendicular to the new capillaries, contributing to increased wound strength [27]. Organized collagen fibers can consistently progress toward normal wound healing [28]. However, in all different treatments in this study, there seems to be no added value in either parameter over the Ringer’s solution treatment used as the control.

This study used two different biofilms, produced with unwashed and washed hydrothermally treated onion pulp, and supplied by BioSmart Nanotechnology. In this process, sliced bulbs were hydrothermally treated in an autoclave for 30 min at 121 °C and 1.2 kgf cm^2^. Onion bulbs were processed through two routes: hydrothermally unwashed and washed pulp. This process modifies the pectin content and structural carbohydrates, leading to bioplastic formation. The carbohydrates and soluble acids identified in the films are glucose, galactose, arabinose, rhamnose, fructose, galacturonic acid, acetic acid, and formic acid. Galacturonic acid comprises approximately 70% of the onion’s pectin [11]. The presence of compounds derived from quercetin is also found in unwashed biofilm. Nevertheless, these films exhibit notable barrier properties, particularly in terms of the fluid handling capacity (FHC), positioning these biopolymers as competitive with top-tier dressings available on the market. Therefore, the key attribute of this biopolymer lies not in the residual presence of phytoncides, but rather in its structural composition. The barrier properties of these biopolymers’ FHC showed potential applicability in food packaging and other areas due to the absence of in vitro mutagenicity and compliance in cytotoxicity assays [10]. Cell viability analysis demonstrated cytotoxicity for unwashed hydrothermally treated pulp than washed hydrothermally treated pulp films, with similar trends for the W-HTP and HTP films sterilized with gamma radiation [29].

Phytoncides, which possess antibiotic properties, have been isolated from various plants, including onions (Allium cepa) [30,31]. These compounds may retain their bactericidal properties even after heat treatment at 100 °C for 20 min (Duka & Ardelean, 2010) [31]. Although this study did not specifically evaluate phytoncides in onion pulp, the results of using onion-based biopolymers on wound healing in horses showed no significant effects.

Several factors may contribute to wound outcomes, including the hydrophilicity of the unwashed and washed hydrothermally treated onion pulp [11], enhancing interaction with the wound bed. Sugars play a crucial role in modulating inflammatory cytokines and exert a chemotactic effect on macrophages, thereby modulating tissue inflammatory response. This modulation promotes the early formation of granulation tissue and accelerates epithelial growth, thus facilitating the wound-healing process [32,33,34]. Applying *Allium cepa* extract, pentaglycan, and allantoin in hypertrophic scars and keloids in human subjects demonstrated an enhancement in the appearance of the scars and keloids, attributed to reduced neoangiogenesis [35].

A murine study assessing the healing effects and properties of flavonoids in an alcoholic extract of *Allium cepa* bulbs demonstrated a pro-healing effect of flavonoids due to their antioxidant activity and free radical elimination [36]. Despite the fact that this study did not evaluate their antibacterial effects, the self-adhesive films containing onion extract have been developed for wound application, releasing effective concentrations of compounds with antioxidant and antibacterial effects against *Staphylococcus epidermidis*, *S. aureus*, and *E. faecalis*, along with anti-inflammatory properties, while preserving cellular viability. In vitro safety assessments conducted with human keratinocytes have demonstrated the onion formulation’s suitability for wound management [37]. Despite all suggested wound healing properties of the unwashed and washed hydrothermally treated onion pulp, this study failed to demonstrate those effects on the healing of skin wounds in horses.

## 5. Conclusions

Properly healing skin wounds in horses remains a significant challenge, driving the continuous search for effective treatments to reduce morbidity and complications. This study, despite conducting thorough gross and histological evaluations, found no substantial positive effects of bacuri butter or onion biopolymers on the healing of skin wounds in horses. However, minor variations observed in the clinical evaluations between treatment groups suggest that further research is needed to fully explore the potential of onion biopolymers and bacuri butter in managing equine skin wounds. Additionally, this study showed the potential of equine models in testing novel therapeutic approaches for wound healing, benefiting both veterinary and human medicine.

## Figures and Tables

**Table 1:** The mean area (cm^2^) and standard deviation (SD) of the wounds in the lumbar region of the horses in the control group (CG) and the groups treated with bacuri butter (BBG), washed onion film (WOFG), and unwashed onion film (UOFG), on days 0 (D0), 3 (D3), 7 (D7), 14 (D14), 21 (D21), and 28 (D28).

Treatment	Wound Area (Mean-cm^2^)												*p*-Value
	D0		D3		D7		D14		D21		D28		
	Mean	SD	Mean	SD	Mean	SD	Mean	SD	Mean	SD	Mean	SD	
GC	10.26 A	0.77	10.38 A	0.82	9.07 aB	0.66	4.24 C	1.57	1.46 D	0.94	0.59 E	0.71	<0.0001
BBG	10.43 AB	1.25	11.09 A	1.24	9.73 abB	1.47	4.63 C	0.71	1.4 D	0.72	0.4 E	0.37	
WOFG	10.67 A	1.45	11.27 A	0.97	10.52 abA	0.8	5.21 B	0.73	2.18 C	0.98	1.47 C	1.31	
UOFG	11.21 A	1.35	11.24 A	1.14	11.32 bA	1.51	4.9 2B	0.49	1.83 C	0.83	0.84 D	0.83	
*p*-Value	ns		ns		0.02		ns		ns		ns		

Different uppercase letters in the same row and different lowercase letters in the same column differ from each other using the Kruskal–Wallis test. (ns: not significant).

**Table 2:** The mean contraction rates, in percentage (%), and standard deviation of the wounds of the horses in the control group (CG) and the groups treated with bacuri butter (BBG), washed onion film (WOFG), and unwashed onion film (UOFG), on days 3 (D3), 7 (D7), 14 (D14), 21 (D21), and 28 (D28).

Treatment	Contraction Rate (%)										*p*-Value
	D3		D7		D14		D21		D28		
	Mean	SD	Mean	SD	Mean	SD	Mean	SD	Mean	SD	
GC	−1.44 A	7.40	10.96 B	11.36	57.85 C	17.1	85.38 D	9.96	93.93 E	7.52	<0.0001
BBG	−8.15 A	22.19	5.04 A	22.48	55.04 B	9.18	86.18 C	8.07	95.95 D	4.00	
WOFG	−6.73 A	12.19	−0.21 A	16.51	50.21 B	10.24	79.60 C	8.23	86.54 C	11.57	
UOFG	−0.59 A	4.62	−1.69 A	9.61	55.54 B	6.69	83.69 C	7.28	92.61 E	7.29	

Different capital letters on the same line differ from each other using the Kruskal–Wallis test.

**Table 3:** The degree of the presence of neutrophils, classified on a scale of absent (−), mild (+), moderate (++), in the wounds of 6 horses in the control group (CG) and groups treated with bacuri butter (BBG), washed onion film (WOFG), and unwashed onion film groups (UOFG), on days 0 (D0), 3 (D3), 7 (D7), 14 (D14), 21 (D21), and 28 (D28).

Evaluated Parameter	Treatment		D0	D3	D7	D14	D21	D28	*p*-Value
Neutrophils	CG	Presence	0/6 (0%)	4/6 (66%)	5/6 (83%)	0/6 (0%)	1/6 (16%)	0/6 (0%)	ns
		Intensity	−	(+)	(+)	−	(+)	−	
	BBG	Presence	0/6 (0%)	6/6 (100%)	2/6 (33%)	0/6 (0%)	0/6 (0%)	0/6 (0%)	ns
		Intensity	−	(++)	(+)	−	−	−	
	WOFG	Presence	0/6 (0%)	6/6 (100%)	5/6 (83%)	0/6 (0%)	0/6 (0%)	0/6 (0%)	ns
		Intensity	−	(++)	(+)	−	(−)	−	
	UOFG	Presence	0/6 (0%)	6/6 (100%)	0/6 (0%)	0/6 (0%)	0/6 (0%)	0/6 (0%)	ns
		Intensity	−	(++)	−	−	−	−	

No difference was observed in Friedman’s chi-square test nor in repeated measures analysis. (ns: not significant).

**Table 4:** The intensity of fibroplasia and the reorganization of collagen fibers within the wounds, classified on a scale of absent (−), mild (+), moderate (++), and severe (+++), in the control group (CG) and groups treated with bacuri butter (BBG), washed onion film (WOFG), and unwashed onion film groups (UOFG), on days 0 (D0), 7 (D7), and 28 (D28).

Evaluated Parameter	TTE	Criterion	D0	D7	D28	*p*-Value
Fibroplasia	GC	Presence	0/6 (0%)	6/6 (100%)	6/6 (100%)	ns
		Intensity	−	(++)	(++)	
	BBG	Presence	0/6 (0%)	6/6 (100%)	6/6 (100%)	ns
		Intensity	−	(+)	(+++)	
	WOFG	Presence	0/6 (0%)	6/6 (100%)	6/6 (100%)	ns
		Intensity	−	(+)	(+++)	
	UOFG	Presence	0/6 (0%)	6/6 (100%)	6/6 (100%)	ns
		Intensity	−	(+)	(++)	
Reorganization of collagen fibers	GC	Presence	0/6 (0%)	6/6 (100%)	6/6 (100%)	ns
		Intensity	−	(++)	(+++)	
	BBG	Presence	0/6 (0%)	6/6 (100%)	6/6 (100%)	ns
		Intensity	−	(+)	(+++)	
	WOFG	Presence	0/6 (0%)	6/6 (100%)	6/6 (100%)	ns
		Intensity	−	(++)	(++)	
	UOFG	Presence	0/6 (0%)	6/6 (100%)	6/6 (100%)	ns
		Intensity	−	(++)	(++)	

No difference was observed in Friedman’s chi-square test nor in repeated measures analysis. (ns: not significant).

## Data Availability

The original contributions presented in the study are included in the article, further inquiries can be directed to the corresponding authors.
